# Olfactory Dysfunction After SARS-CoV-2 Infection in the RECOVER Adult Cohort

**DOI:** 10.1001/jamanetworkopen.2025.33815

**Published:** 2025-09-25

**Authors:** Leora I. Horwitz, Jacqueline H. Becker, Weixing Huang, Teresa Akintonwa, Maxwell M. Hornig-Rohan, Gabrielle Maranga, Dara R. Adams, Mark W. Albers, Mirna Ayache, Jasmine Berry, Hassan Brim, Tanner W. Bryan, Alexander W. Charney, Robert A. Clark, Melissa M. Cortez, Brian D’Anza, Hannah Davis, Sarah E. Donohue, Nathaniel Erdmann, Valerie Flaherman, Tamara G. Fong, Jennifer A. Frontera, Mark P. Goldberg, Jason D. Goldman, Michelle S. Harkins, Sally L. Hodder, Vanessa L. Jacoby, Prasanna Jagannathan, Xiaolin Jia, John Daniel Kelly, Jerry A. Krishnan, Andre Kumar, Adeyinka O. Laiyemo, Emily B. Levitan, Jeffrey N. Martin, Kathryn M. McCaffrey, Grace A. McComsey, Torri D. Metz, Ganesh Murthy, Helen Nguyen, Megumi Okumura, Samuel Parry, Sairam Parthasarathy, Thomas F. Patterson, Michael J. Peluso, Christina Sorochinsky, Tiffany Walker, Samantha L. Wiegand, Zanthia Wiley, Juan Wisnivesky, Hassan Ashktorab, Andrea Foulkes, Joyce K. Lee-Iannotti

**Affiliations:** 1 Department of Population Health, NYU (New York University) Grossman School of Medicine, New York; 2 Department of Medicine, NYU Grossman School of Medicine, New York; 3 Department of Medicine, Icahn School of Medicine at Mount Sinai, New York, New York; 4 Massachusetts General Hospital Biostatistics, Boston; 5 RECOVER (Researching COVID to Enhance Recovery) Patient, Caregiver, or Community Advocate Representative, New York, New York; 6 Illinois Research Network and Department of Otolaryngology-Head and Neck Surgery, University of Illinois, Chicago, College of Medicine, Chicago; 7 Department of Neurology, Massachusetts General Hospital, Harvard Medical School, Boston; 8 Department of Pulmonary, Critical Care, and Sleep Medicine, MetroHealth Medical Center of Case Western Reserve University, Cleveland, Ohio; 9 Department of Infectious Disease, Emory University School of Medicine, Atlanta, Georgia; 10 Department of Medicine, Howard University College of Medicine, Washington, DC; 11 Public Health Institute, Denver Health, Denver, Colorado; 12 Icahn School of Medicine at Mount Sinai, New York, New York; 13 Institute for Integration of Medicine & Science, University of Texas Health Science Center, San Antonio; 14 Department of Neurology, University of Utah, Salt Lake City; 15 Division of Rhinology and Skull Base Surgery, Department of Otolaryngology, Head and Neck Surgery, Case Western Reserve University, University Hospitals Cleveland Medical Center, Cleveland, Ohio; 16 Patient-Led Research Collaborative, New York, New York; 17 Illinois Research Network and Department of Psychiatry and Behavioral Medicine, University of Illinois College of Medicine Peoria, Peoria; 18 Department of Medicine, University of Alabama at Birmingham; 19 Institute for Health Policy Studies, University of California, San Francisco, San Francisco; 20 Department of Neurology, Beth Israel Deaconess Medical Center, Harvard Medical School, Boston, Massachusetts; 21 Department of Neurology, NYU Grossman School of Medicine, New York; 22 Department of Neurology, University of Texas Health San Antonio; 23 Providence Swedish Medical Center, Seattle, Washington; 24 Division of Allergy and Infectious Diseases, University of Washington, Seattle; 25 Department of Medicine, University of New Mexico, Albuquerque; 26 Department of Medicine, West Virginia University, Morgantown; 27 Department of Obstetrics, Gynecology, and Reproductive Sciences, University of California, San Francisco, San Francisco; 28 Department of Medicine, Stanford University School of Medicine, Stanford, California; 29 Department of Medicine, San Francisco Veterans Affairs Medical Center, University of California, San Francisco, San Francisco; 30 Illinois Research Network and Department of Medicine, University of Illinois Chicago, Chicago; 31 Department of Epidemiology, University of Alabama at Birmingham; 32 Department of Epidemiology and Biostatistics, University of California, San Francisco, San Francisco; 33 Department of Medicine, Division of Allergy and Infectious Disease, University of Washington, Seattle; 34 Division of Infectious Diseases, Departments of Medicine and Pediatrics, Case Western Reserve University, University Hospitals Cleveland Medical Center, Cleveland, Ohio; 35 Department of Obstetrics and Gynecology, University of Utah Health, Salt Lake City; 36 Department of Neurology, University of Arizona College of Medicine–Phoenix; 37 Department of Pediatrics, University of California, San Francisco; 38 Department of Internal Medicine, University of California, San Francisco; 39 PRL Institute for Health Policy Studies, University of California, San Francisco; 40 Department of Obstetrics and Gynecology, University of Pennsylvania, Philadelphia; 41 University of Arizona Health Sciences Center for Sleep Circadian and Neuroscience Research, Tucson; 42 Department of Medicine and Infectious Diseases, University of Texas Health San Antonio; 43 Division of HIV, Infectious Diseases, and Global Medicine, University of California, San Francisco; 44 Department of Medicine, Emory University, Atlanta, Georgia; 45 Maternal-Fetal Medicine, Miami Valley Hospital, Dayton, Ohio; 46 Division of Infectious Diseases, Department of Medicine, Emory University, Atlanta, Georgia; 47 Division of General Internal Medicine, Icahn School of Medicine at Mount Sinai, New York, New York; 48 Harvard Medical School, Harvard University, Boston, Massachusetts; 49 Department of Biostatistics, T. H. Chan School of Public Health, Harvard University, Boston, Massachusetts

## Abstract

**Importance:**

Olfactory dysfunction is common after SARS-CoV-2 infection and has been associated with cognitive loss in other conditions. Formal testing is needed to characterize the presence, severity, and patterns of olfactory dysfunction.

**Objective:**

To characterize long-term olfactory dysfunction after SARS-CoV-2 infection.

**Design, Setting, and Participants:**

This prospective cohort study included adults enrolled in the Researching COVID to Enhance Recovery (RECOVER)–Adult study. All those with and a random sample of those without self-reported change or loss in smell or taste were offered olfactory testing, performed at 83 sites in 35 US states and territories. Participants included 2956 enrollees with prior infection (1393 with and 1563 without self-reported change or loss) and 569 without prior infection (9 with and 560 without self-reported change or loss in taste) who underwent olfactory testing a mean (SD) of 671.6 (417.8) days after the index date. Data were collected from October 29, 2021, to June 6, 2025.

**Exposure:**

SARS-CoV-2 infection.

**Main Outcomes and Measures:**

Olfactory function, as defined by age- and sex-standardized performance on the University of Pennsylvania Smell Identification Test (UPSIT), a well-validated test comprising 40 unique odors.

**Results:**

The study included 3525 participants with a mean (SD) age of 47.6 (15.2) years; of 3520 with data available, 2548 (72.4%) were female or intersex. Among 1393 infected participants with self-reported change or loss, 1111 (79.8%) had hyposmia on the UPSIT, including 321 (23.0%) with severe microsmia or anosmia. Among 1563 infected participants without self-reported change or loss, 1031 (66.0%) had hyposmia, including 128 (8.2%) with severe microsmia or anosmia. Participants with prior infection and self-reported change or loss scored at the 16th age- and sex-standardized UPSIT percentile, compared with the 23rd and 28th percentiles for those without self-reported change or loss with and without prior known infection, respectively. Younger women had scores corresponding to lower mean age- and sex-standardized percentiles. Among participants who self-reported change or loss in smell, those with abnormal UPSIT scores more often reported cognitive problems (742 of 1111 [66.8%]) than those with normal UPSIT scores (179 of 282 [63.5%]).

**Conclusions and Relevance:**

In this cohort study of RECOVER-Adult participants, self-reported change or loss in smell or taste was an accurate signal of verified hyposmia, but a high rate of hyposmia among those with no reported change or loss was also observed. Formal smell testing may be considered in those with prior SARS-CoV-2 infection to diagnose occult hyposmia and counsel patients about risks.

## Key Points

**Question:**

What are patterns of olfactory dysfunction in adults after SARS-CoV-2 infection?

**Findings:**

In this cohort study, 1111 of 1393 SARS-CoV-2–infected participants who reported loss in or change of smell or taste a mean of 2 years after infection (80%) had hyposmia on formal testing, a total of 321 (23%) had severe microsmia or anosmia, and the mean age- and sex-standardized score was at the 16th percentile. Hyposmia was also present in 1031 of 1563 participants (66%) with prior infection but no self-reported change or loss (mean: 23rd percentile).

**Meaning:**

These findings suggest that occult hyposmia following infection with SARS-CoV-2 is common, and olfactory testing should be considered after infection to diagnose olfactory dysfunction and counsel patients about the risks of smell loss.

## Teaser

This cohort study examines the presence, severity, and patterns of olfactory dysfunction in US adults with and without prior SARS-CoV-2 infection.

## Introduction

Self-reported loss of or change in the sense of smell is a cardinal manifestation of SARS-CoV-2 infection, seen in approximately 80% of people with acute infection in the original and Alpha waves of the pandemic and one-third of patients following infection with Omicron variants.^1,2^ Beyond variant type, other factors associated with loss of smell at the time of initial infection include female sex,^3^ use of e-cigarettes,^3^ Hispanic ethnicity,^3,4,5^ and non-Hispanic Black race.^4^ Black or African American and non-Mexican Hispanic persons were more likely to report recovery of smell.^3^

Loss of or change in smell and taste can persist for months or years^6,7^ and has important consequences, including weight loss,^8^ reduction in social interaction and quality of life,^9,10^ and safety risks of being unable to identify spoiled food, gas leaks, smoke, and other dangers.^9,11^ Additionally, epidemiological studies have linked impaired olfaction to neurodegenerative diseases, many of which involve pathophysiological changes in the brain’s olfactory regions.^12,13^ Decades of research have found that verified olfactory dysfunction is a strong early factor associated with neurodegenerative disease, often preceding diagnosis by years.^14^ Cognition and olfactory function are intricately linked. The olfactory system is closely connected to brain areas involved in memory, emotion, and decision-making. Viruses may enter the brain directly through the nasal epithelium and cause neuroinflammation and abnormal protein aggregation in addition to olfactory damage.^15^

Whether olfactory dysfunction following SARS-CoV-2 infection will lead to cognitive deficits is uncertain. One UK Biobank study of participants with pre– and post–SARS-CoV-2 infection brain magnetic resonance imaging found that people with prior SARS-CoV-2 infection had evidence of greater tissue damage in the primary olfactory cortex, greater reduction in gray matter thickness in the orbitofrontal cortex and parahippocampal gyrus, and greater reduction in global brain size than those without infection.^16^ However, despite the high prevalence of self-reported impairments in smell in SARS-CoV-2 infection, few studies have explored impairments using validated tools.^1,17,18,19^ Given that self-reported smell and taste function do not consistently correlate with formal testing,^20,21^ formal testing is necessary to identify persistence, severity, and patterns of smell loss.

Accordingly, leveraging data from the National Institutes of Health–funded Researching COVID to Enhance Recovery (RECOVER)–Adult study, we examined our primary outcome of olfactory function, specifically (1) the degree to which people with prior SARS-CoV-2 infection and self-reported change or loss in smell have abnormal performance on the University of Pennsylvania Smell Identification Test (UPSIT); (2) whether those without self-reported change have occult impairments in olfactory function; and (3) whether there are any specific patterns of changes in sense of smell in SARS-CoV-2. Secondarily, we explored whether olfactory dysfunction is associated with self-reported cognitive impairment as measured by a validated instrument (the Neuro-QoL).

## Methods

### Study Population and Data Sources

All participants provided written informed consent. The study was approved by the NYU Grossman School of Medicine Institutional Review Board and followed the Strengthening the Reporting of Observational Studies in Epidemiology (STROBE) reporting guideline for cohort studies.

The RECOVER-Adult study recruited adults 18 years or older with and without history of SARS-CoV-2 infection and followed them up prospectively with symptom surveys approximately every 90 days, beginning October 29, 2021. These analyses leveraged data collected through June 6, 2025. Participants enrolling as uninfected had confirmatory nucleocapsid antibody and SARS-CoV-2 antigen testing on enrollment and were reclassified as infected if the results were positive. The index date for infected participants was the date of the first infection, and for uninfected participants was the date of a negative test result. Participants were asked whether they experienced any “change in or loss of smell or taste” (hereinafter referred to as self-reported change or loss). At each visit at least 3 months after infection, participants with prior infection who answered affirmatively were offered the opportunity to take the UPSIT, as were a randomly selected 15% of participants who answered negatively. Accordingly, there were 4 analytic groups: those with prior infection with and without self-reported change or loss and those without prior infection with and without self-reported change or loss. Participants were assigned to analytic groups according to infection status and self-reported change or loss status within 90 days of completing the UPSIT. Thus, participants who enrolled as uninfected but had positive findings for infection during the study and then underwent UPSIT evaluation were included in the previously infected group. Participants with earlier self-reported change or loss but no reported change or loss at the time of testing were included in the group with no self-reported change or loss. Participants who did not undergo the UPSIT evaluation the first time it was offered were offered the evaluation at subsequent visit(s) if still self-reporting change or loss. Participants who reported cognitive dysfunction, chronic sinusitis, or loss of smell or taste before the index date were excluded, as were participants who did not answer every UPSIT question.

### Measures

Our primary outcome was olfactory function, defined as a binary variable of normal on UPSIT testing vs not. In secondary analyses, we assessed the UPSIT score as a continuous variable, and as an age- and sex-normed percentile. The UPSIT is a well-established, highly reliable 40-item scratch and sniff multiple-choice test (4 response options per odor) in which participants are required to answer every question even if they cannot discern an odor.^22,23,24^ Because of baseline differences between olfactory function in male and female patients, the test is differentially scored by sex. Each correct answer receives 1 point. Scores of 34 to 40 in men and 35 to 40 in women are defined as normal. Scores of 30 to 33 in men and 31 to 34 in women are defined as mild microsmia; 26 to 29 in men and 26 to 30 in women, as moderate microsmia; 19 to 25 in both sexes, as severe microsmia; and less than 19 in both sexes, as anosmia. Scores of 5 or less are noted by the developer to be statistically improbable, even in people with total anosmia, given the requirement for guessing an answer on every question (probability ≤5, 4.3%) and we therefore reported these separately but still considered them abnormal for the primary outcome. Age-and sex-stratified percentile norms are provided by UPSIT developers based on prepandemic data. For subanalyses involving specific odors, we grouped smells into pleasant, neutral, and unpleasant categories as defined by empiric work by the developers because prior studies have suggested an association of unpleasant smell loss with Parkinson disease and other neurodegenerative disorders.^25,26^

For the secondary objective of assessing association of cognition with olfactory dysfunction, we used self-reported problems thinking or concentrating (brain fog), which was assessed in all participants. Those study participants self-reporting brain fog also received the Neuro-QoL Short Form, version 2.0 Cognitive Function instrument, a self-reported 8-item assessment of cognitive function that is nationally normed to have a median T score of 50 and SD of 10.^27^

### Statistical Analysis

Cohort characteristics (demographic characteristics, enrollment factors, and vaccination status at index) were summarized, stratified by self-reported change or loss and infection status using counts and relative frequencies for categorical variables and mean (SD) or median (IQR) for quantitative variables. Race and ethnicity were self-reported by participants based on categories used in the All of Us program so that we could characterize demographics. For reporting purposes, we combined American Indian or Alaska Native, and Native Hawaiian or Other Pacific Islander into an other category. UPSIT findings, including normal vs abnormal status, raw UPSIT score, and UPSIT percentiles adjusted by age and sex, were also summarized by self-reported change or loss and infection status.

The proportion of participants answering each UPSIT question correctly was summarized using a heatmap stratified by self-report, infection status, and microsomia status. To study patterns of olfactory dysfunction among participants with an abnormal UPSIT score, proportions of participants answering each UPSIT question correctly by infection status and self-report were calculated by UPSIT smell category (pleasant, neutral, and unpleasant, as defined by the UPSIT developers^23^) using forest plots. In this unadjusted and exploratory analysis, 95% CIs were reported without an adjustment for multiple testing.

K-means consensus clustering was performed to group participants with history of infection, self-report of change or loss, and abnormal UPSIT scores into clusters exhibiting similar patterns of olfactory dysfunction.^28^ Responses to 40 UPSIT questions were used as input, and a heatmap was used to illustrate cluster characteristics. This data-based strategy permits the identification of distinct olfactory profiles without imposing predefined groups, thereby capturing the inherent heterogeneity of smell loss. By aggregating across multiple iterations of the K-means algorithm, consensus clustering enhances the stability of the clustering outcome, addressing the sensitivity of conventional K-means clustering to initial conditions. Means and SDs were reported for Neuro-QoL cognitive score among participants completing the Neuro-QoL assessment by self-reported change or loss and microsomia status.

Statistical analyses were performed using the ConsensusClusterPlus package of R Software, version 4.4.0 (R Program for Statistical Computing), for cluster analysis. All study data were stored in a Research Electronic Data Capture (REDCap) database housed in a FISMA (Federal Information Security Modernization Act) moderate compliant environment.

## Results

### Study Population

Of 15 157 participants enrolled in the RECOVER adult study, we included 3525 participants, consisting of 2956 with prior infection at time of testing (1393 with self-reported change or loss) and 569 with no infection (9 with self-reported change or loss). Participants underwent UPSIT evaluation within 90 days of a symptom survey (Figure).

**Figure.**
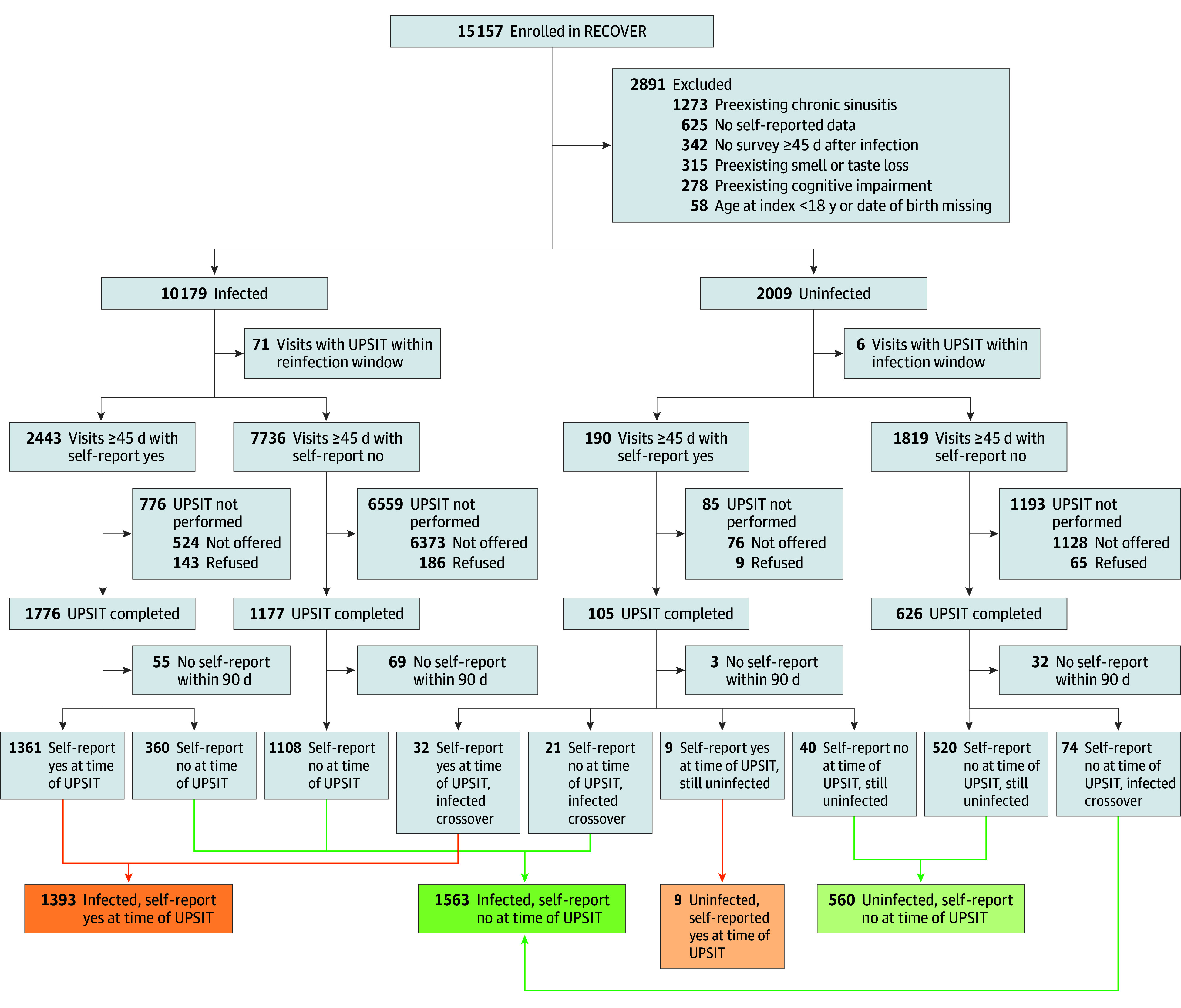
Flow Diagram of Participants Included in Analysis Self-report yes or no indicates self-reported change in or loss of smell or taste. RECOVER indicates Researching COVID to Enhance Recovery; UPSIT, University of Pennsylvania Smell Identification Test.

Demographic characteristics of the analytic cohort are shown in Table 1. Study participants had a mean (SD) age of 47.6 (15.2) years; among the 3520 with data available, 2548 (72.4%) were female or intersex and 972 (27.6%) were male. The interval from index date to UPSIT was a mean (SD) of 671.6 (417.8) days (1.8 years) overall and 742.6 (417.6) days (2.0 years) among those with prior infection and self-reported change or loss. Characteristics of participants who did and did not complete the UPSIT (stratified by infection status) are shown in eTable 1 in Supplement 1; there were no substantive differences.

**Table 1.** Demographic Characteristics of Participants Completing UPSIT, by Infection and Symptom Status

Characteristic	Participant category, No. (%)^a^				
	Prior infection and self-reported yes (n = 1393)	Prior infection and self-reported no (n = 1563)	No infection and self-reported yes (n = 9)	No infection and self-reported no (n = 560)	Overall (N = 3525)
Age at index, y					
Mean (SD)	47.5 (14.7)	46.5 (15.6)	50.3 (15.8)	50.6 (15.1)	47.6 (15.2)
Median (IQR)	47.1 (35.2-58.7)	44.8 (33.1-59.3)	48.8 (39.2-62.9)	52.3 (38.0-62.5)	47.1 (34.6-59.9)
Age category at index, y					
18-45	627 (45.0)	784 (50.2)	4 (44.4)	211 (37.7)	1626 (46.1)
46-65	575 (41.3)	542 (34.7)	3 (33.3)	246 (43.9)	1366 (38.8)
>65	191 (13.7)	237 (15.2)	2 (22.2)	103 (18.4)	533 (15.1)
Sex assigned at birth					
Female or intersex^b^	1076 (77.5)	1095 (70.1	7 (77.8)	370 (66.1)	2548 (72.4)
Male	313 (22.5)	467 (29.9)	2 (22.2)	190 (33.9)	972 (27.6)
No. missing	4	1	0	0	5
Self-reported race and ethnicity					
Hispanic	293 (21.2)	285 (18.4)	1 (11.1)	75 (13.4)	654 (18.7)
Non-Hispanic Asian	44 (3.2)	81 (5.2)	1 (11.1)	37 (6.6)	163 (4.7)
Non-Hispanic Black	164 (11.8)	214 (13.8)	1 (11.1)	91 (16.3)	470 (13.4)
Non-Hispanic White	831 (60.0)	907 (58.4)	6 (66.7)	333 (59.7)	2077 (59.3)
Multiracial or other^c^	52 (3.8)	65 (4.2)	0	22 (3.9)	139 (4.0)
No. missing	9	11	0	2	22
Enrollment cohort and era					
Pre-Omicron	787 (56.5	484 (31.0)	1 (11.1)	112 (20.0)	1384 (39.3)
Acute Omicron	300 (21.5)	588 (37.6)	3 (33.3)	257 (45.9)	1148 (32.6)
Postacute Omicron	306 (22.0)	491 (31.4)	5 (55.6)	191 (34.1)	993 (28.2)
Vaccinated at first infection					
Unvaccinated	696 (506)	463 (30.0)	1 (11.1)	73 (13.3)	1233 (35.5)
Partially vaccinated or date of last dose unknown	52 (3.8)	47 (3.0)	0	20 (3.6)	119 (3.4)
Fully vaccinated	627 (45.6)	1033 (66.9)	8 (88.9)	455 (83.0)	2123 (61.1)
No. missing	18	20	0	12	50
Acute hospitalization					
Not hospitalized during acute phase	1175 (87.9)	1344 (91.5)	NA	NA	2519 (89.8)
Hospitalized during acute phase	162 (12.1)	125 (8.5)	NA	NA	287 (10.2)
No. missing	56	94	9	560	719
Household income, US $					
<25 000	218 (17.3)	234 (16.5)	4 (44.4)	106 (21.0)	562 (17.6)
25 000-49 999	238 (18.9)	208 (14.7)	0	64 (12.7)	510 (16.0)
≥$50 000	803 (63.8)	977 (68.9)	5 (55.6)	335 (66.3)	2120 (66.4)
No. missing	134	144	0	55	333
Rural or urban					
Not rural	1290 (92.6)	1501 (96.0)	6 (66.7)	541 (96.6)	3338 (94.7)
Rural	103 (7.4%	62 (4.0)	3 (33.3)	19 (3.4)	187 (5.3)
Educational attainment					
Bachelor’s or advanced degree	767 (57.5)	1025 (68.0)	7 (77.8)	378 (71.6)	2177 (64.4)
High school, GED, some college, vocational, or technical	568 (42.5)	482 (32.0)	2 (22.2)	150 (28.4)	1202 (35.6)
No. missing	58	56	0	32	146
Time from index to UPSIT, d					
Mean (SD)	742.6 (417.6)	657.3 (421.1)	591.3 (302.8)	535.9 (371.3)	671.6 (417.8)
Median (IQR)	725.0 (393.0- 1038.0)	583.0 (317.5- 987.0)	523.0 (322.0- 757.0)	455.0 (200.0- 753.0)	637.0 (334.0- 981.0)

Abbreviations: GED, general educational development; NA, not applicable; UPSIT, University of Pennsylvania Smell Identification Test.

^a^ Self-report yes or no indicates yes or no self-reported change in or loss of smell or taste.

^b^ Results are not reported for groups with fewer than 5 participants; therefore female and intersex have been combined.

^c^ Includes American Indian or Alaska Native and Native Hawaiian or Other Pacific Islander.

### Association Between Self-Reported Olfactory Dysfunction and UPSIT

Among the 1393 infected participants with self-reported change or loss, 1111 (79.8%; positive predictive value, 79.8% [95% CI, 77.5%- 81.8%]) had an abnormal UPSIT score (hyposmia). A total of 321 (23.0%) had severe microsmia or anosmia (Table 2). Among the 560 uninfected participants without self-reported change or loss, 336 (60.0%) had hyposmia, including 52 (9.3%) with severe microsmia or anosmia. Among the 1563 participants with prior infection and without self-reported change or loss, 1031 (66.0%) had hyposmia, including 128 (8.2%) with severe microsmia or anosmia. By contrast, 532 of the 1563 participants (34.0%) with prior COVID-19 infection without self-reported change or loss had a normal UPSIT score (negative predictive value, 34.0% [95% CI, 31.7%-36.5%]), as did 224 of 560 (40.0%) without prior infection and without self-reported change or loss (negative predictive value, 40.0% [95% CI, 35.9%-44.2%]). The raw UPSIT score tended to be lower in infected participants with vs without self-reported change or loss (median score, 30.0 [IQR, 26.0-34.0] vs 33.0 [IQR, 30.0-35.0]) (Table 2). The distribution of age- and sex-standardized UPSIT percentiles tended to be lower in infected participants with self-reported change or loss (mean [SD]: 16th [21st] percentile) compared with infected (mean [SD]: 23rd [22nd] percentile) and uninfected (mean [SD]: 28th [24th] percentile) participants without self-reported change or loss and were on average worse in women and those aged 18 to 45 years (eFigure 1 and eTable 2 in Supplement 1).

**Table 2.** UPSIT Findings by self-Report, Stratified by Infection Status

Finding	Self-reported loss or change in smell or taste, No. (%)			
	Infected		Uninfected	
	Yes (n = 1393)	No (n = 1563)	Yes (n = 9)	No (n = 560)
UPSIT findings				
Normal	282 (20.2)	532 (34.0)	3 (33.3)	224 (40.0)
Mild microsmia	420 (30.2)	615 (39.3)	1 (11.1)	203 (36.3)
Moderate microsmia	359 (25.8)	288 (18.4)	1 (11.1)	81 (14.5)
Severe microsmia	194 (13.9)	102 (6.5)	2 (22.2)	35 (6.3)
Anosmia	127 (9.1)	26 (1.7)	2 (22.2)	17 (3.0)
Score <6	11 (0.8)	0	0	0
Microsomia status				
Normal	282 (20.2)	532 (34.0)	3 (33.3)	224 (40.0)
Abnormal	1111 (79.8)	1031 (66.0)	6 (66.7)	336 (60.0)
UPSIT score^a^				
Mean (SD)	28.8 (6.9)	32.0 (4.5)	26.9 (9.4)	32.1 (.0)
Median (IQR)	30.0 (26.0-34.0)	33.0 (30.0-35.0)	28.0 (21.0-35.0)	33.0 (30.0-35.3)
UPSIT percentile adjusted by age and sex				
Mean (SD)	15.7 (20.5)	22.5 (22.0)	22.6 (31.5)	27.9 (23.8)
Median (IQR)	9.0 (0.0-22.0)	16.5 (6.0-33.0)	0 (0-59.0)	21.0 (9.0-42.3)
No. missing	4	1	0	0

Abbreviation: UPSIT, University of Pennsylvania Smell Identification Test.

^a^ Scores of 34 to 40 in men and 35 to 40 in women are defined as normal. Scores of 30 to 33 in men and 31 to 34 in women are defined as mild microsmia; 26 to 29 in men and 26 to 30 in women, as moderate microsmia; 19 to 25 in both sexes, as severe microsmia; and less than 19 in both sexes, as anosmia. Scores of 5 or less are noted by the developer to be statistically improbable.

### Patterns of Olfactory Dysfunction

Among participants with an abnormal UPSIT score, in unadjusted analysis, patterns of smell loss were similar between infected and uninfected participants without self-reported change or loss. By contrast, infected participants with an abnormal UPSIT score and self-reported change or loss had relatively worse detection of smells in all categories: pleasant, neutral, and unpleasant (eFigure 2 in Supplement 1). The most marked differences included detection of cloves (correct responders: 764 of 1111 [68.8%] infected with self-reported change or loss vs 832 of 1031 [80.7%] infected without and 280 of 336 [83.3%] uninfected without), grass (correct responders: 822 of 1111 [74.0%] infected with self-reported change or loss vs 892 of 1031 [86.5%] infected without and 293 of 336 [87.2%] uninfected without), licorice (correct responders: 769 of 1111 [69.2%] infected with self-reported change or loss vs 852 of 1031 [82.6%] infected without and 275 of 336 [81.8%] uninfected without), and watermelon (correct responders: 605 of 1111 [54.5%] infected with self-reported change or loss vs 687 of 1031 [66.6%] infected without and 226 of 336 [67.3%] uninfected without). Proportions answering each question correctly among participants with normal UPSIT scores are shown in eFigure 3 in Supplement 1. As these results are not adjusted for demographic differences across groups, they are considered exploratory.

Infected participants with self-reported change or loss and abnormal UPSIT scores were classified into 4 clusters with distinct patterns of smell loss (eFigure 4 in Supplement 1). Cluster 1 (n = 358) had isolated citrus (lime and lemon) loss; cluster 2 (n = 389), turpentine loss; cluster 3 (n = 225), moderate loss, predominantly citrus, watermelon, cedar, licorice, and pizza; and cluster 4 (n = 139) extensive loss, with greatest loss for fruit punch and bubble gum. Overall UPSIT scores were lower in clusters 3 and 4 (median, 23 [IQR, 21-25] and 14 [IQR, 11-16], respectively) compared with clusters 1 and 2 (median, 31 [IQR, 29-33] and 31 [IQR, 29-32], respectively) (eFigure 5 and eTable 3 in Supplement 1). Infected participants with self-reported change or loss and normal UPSIT scores (n = 282) were only classified into cluster 1 (208 [73.8%]) and cluster 2 (74 [26.2%]).

### Association Between Olfactory Dysfunction and Neuro-QoL

Among 2956 infected participants, 1432 self-reported problems with thinking or concentrating, triggering the automatic addition of Neuro-QoL questions. Among infected participants with self-reported change or loss, 921 of 1393 (66.1%) self-reported problems with thinking, compared with only 511 of 1563 (32.7%) of participants without change or loss (Table 3). Frequency of these self-reported cognitive problems was lower among those with self-reported change or loss who had normal UPSIT scores (179 of 282 [63.5%]) compared with those who had abnormal UPSIT scores (742 of 1111 [66.8%). Among participants undergoing UPSIT who self-reported brain fog, the mean (SD) Neuro-QoL score was similar in those with self-reported change or loss and abnormal UPSIT scores (T score, 38 [8]; 12th percentile) compared with normal UPSIT scores (T score, 39 [9]; 14th percentile). Similarly, in participants without self-reported change or loss, Neuro-QoL scores were similar between those with abnormal and normal UPSIT scores (mean [SD] T score, 42 [7] for both) (Table 3).

**Table 3.** Cognitive Function Among Infected Participants With and Without Self-Reported Loss

Characteristic	Self-reported loss of or change in smell or taste			
	Yes		No	
	Problems with thinking, No./total No. (%)	Neuro-QoL T score, mean (SD)^a^	Problems with thinking, No./total No. (%)	Neuro-QoL T score, mean (SD)^a^
All ages	921/1393 (66.1)	38 (8)	511/1563 (32.7)	42 (7)
Age 18-45 y	421/627 (67.1)	38 (8)	287/784 (36.6)	42 (7)
Age 46-65 y	413/575 (71.8)	37 (8)	181/542 (33.4)	42 (7)
Age >65 y	87/191 (45.5)	41 (8)	43/237 (18.1)	43 (6)
Abnormal UPSIT	742/1111 (66.8)	38 (8)	333/1031 (32.3)	42 (7)
Normal UPSIT	179/282 (63.5)	39 (9)	178/532 (33.5)	42 (7)

Abbreviation: UPSIT, University of Pennsylvania Smell Identification Test.

^a^ Among those reporting problems thinking. This self-reported 8-item assessment of cognitive function is nationally normed to have a median T score of 50 and SD of 10.^27^

## Discussion

In this cohort study of 3525 participants undergoing formal testing of 40 different smells after SARS-CoV-2 infection using the UPSIT tool a mean of 1.8 years after index date, we found that self-reported change in or loss of smell or taste in this population accurately reflected olfactory dysfunction: 79.8% with self-reported change or loss had hyposmia on UPSIT. On average, participants with prior SARS-CoV-2 infection and self-reported change or loss had UPSIT scores at the 15th percentile for their age and sex. However, 66.0% of infected participants without self-reported change or loss also had abnormal UPSIT scores (as did 60.0% without prior infection and no self-reported change or loss), suggesting unrecognized olfactory loss is both common in the general population and more prevalent among those with prior infection. Abnormal UPSIT scores coincided with self-reported cognitive deficits.

Our findings corroborate prior survey studies suggesting that SARS-CoV-2 is associated with persistent olfactory dysfunction^29^ and confirm small prior objective studies finding that patients underestimate their smell loss.^30^ The reason for underestimation is uncertain. It is possible that cognitive deficits could contribute to decreased awareness of sensory changes. One study, for instance, found that patients with persistent olfactory dysfunction after SARS-CoV-2 infection had lower gray matter volumes both in the olfactory cortex and in other brain regions related to cognitive, sensory, and emotion processing than those without olfactory dysfunction.^31^ Conversely, olfactory dysfunction preceding cognitive dysfunction and neurodegenerative disease is well recognized in the prepandemic literature.^14,32,33,34^ Historically, postviral olfactory dysfunction has not been considered to carry the same risk for cognitive dysfunction as age-associated or neurodegenerative disease–associated olfactory dysfunction, but large-scale data on this question are lacking. Future research should explore the longitudinal relationship of self-reported change or loss or objective measures of olfactory dysfunction such as UPSIT with subsequent neurological decline.^35^

Our study involved participants with persistent deficits at a mean of 1.8 years from infection, among whom we found differentially worse than expected performance in younger women compared with historical norms; in contrast, another study found that younger people were more likely to recover within 6 months after infection.^36^ Additionally, we identified specific smells and groups of smells affected. The most marked individual smell differences between people with self-reported change or loss and those without spanned multiple domains: watermelon (pleasant), cloves and grass (neutral), and licorice (unpleasant), without apparent preference for one domain over another. By contrast, Parkinson disease has been reported to differentially affect the unpleasant domain,^37,38,39^ likely due to selective neurodegeneration in the amygdala and piriform cortex.^40^ This may help differentiate causes of olfactory dysfunction in patients with abnormal UPSIT scores and suggests that different brain regions are impacted by SARS-CoV-2. Infected participants with the lowest UPSIT scores were generally grouped in cluster 4, which had widespread deficits but worse performance in pleasant smells (eg, fruit punch, bubble gum). Prior studies have shown that pleasant odors are processed elsewhere, in the orbitofrontal cortex and ventral striatum.^40^

We also found that detection of citrus smells—lemon in particular—was lower than other odorants for both infected and uninfected participants. At least 2 studies have similarly found that lemon is the most incorrectly detected scent in patients after COVID-19 infection and in uninfected controls.^17,41,42^ Whether this is a true finding or related to the UPSIT in particular requires further investigation.

The UPSIT specifically measures odor identification. Other options exist for formal olfactory testing. In addition to odor identification, the Sniffin’ Sticks test^43^ assesses olfactory discrimination (ability to distinguish between odors) and threshold (the lowest concentration of an odorant that can be reliably detected). The Connecticut Chemosensory Clinical Research Center Test is another olfactory threshold test.^44^ Electro-olfactography^45^ and olfactory-evoked potentials^46^ measure brain activity in response to odors. Use of these tests, while less accessible in clinical practice, might uncover additional post–SARS-CoV-2 olfactory dysfunction of interest.

Treatments for smell loss after SARS-CoV-2 infection, such as olfactory training, have shown some promise in facilitating recovery.^47,48^ Olfactory training encourages gradual recovery by repeated exposure to specific odors to stimulate neural pathways and is included in consensus guidelines for treatment.^49^ Platelet-rich plasma injections into the olfactory cleft are being studied with some early promising results. Adjunctive treatments have been explored with varying success, such as intranasal application of corticosteroids to reduce inflammation; sodium citrate, which may modulate olfactory signaling; and vitamin A supplementation, believed to support mucosal repair.^50,51^ To the extent that cognitive dysfunction contributes to unreported smell loss, cognitive rehabilitation might be useful. A deeper understanding of SARS-CoV-2’s impact on sensory systems and cognition may aid in refining these therapies.

Our study is substantially larger than other studies using formal testing^1,17,18,31,52,53^ and includes individuals without self-reported smell loss and uninfected individuals for comparison, while excluding those with pre-existing comorbidities known to affect olfactory function such as chronic sinusitis and cognitive impairment. The UPSIT, a validated and objective measure of olfactory dysfunction, enables analysis of abnormal smell perception patterns.

### Limitations

Limitations include the lack of assessment for phantom smells (phantosmia)^54^ and the omission of assessment of taste loss, which often accompanies olfactory loss. We did not have data on pre-existing head trauma, which can result in olfactory dysfunction, although we do not expect it to be common. The Neuro-QoL score was only obtained in those reporting cognitive impairment, limiting assessments of that outcome. It is likely that some infected and uninfected individuals were misclassified given lack of universal testing and potential for asymptomatic infections. The surprisingly high rate of hyposmia among putatively uninfected individuals, for instance, may indicate asymptomatic infection in this group. Most participants were tested after many months of persistent symptoms, making this group not representative of those with early, transient olfactory loss. Infected and uninfected participants had slightly different demographic characteristics, which is why we used age- and sex-standardized measures. Finally, test administration error (eg, overscratching cards) may have contributed to some incorrect answers, leading to an overestimation of smell loss, although that would be expected to affect all participants similarly.

## Conclusions

In this cohort study, we found a high burden of persistent olfactory dysfunction after SARS-CoV-2 infection, even among those not reporting concerns. Given the degree of hyposmia in persons previously infected, formal olfactory testing may be beneficial in standard postinfection care.^55^ The temporal associations of cognitive dysfunction with olfactory dysfunction after SARS-CoV-2 infection will need further investigation.

## Notes

Supplement 1. **eFigure 1.** Distribution of Age- and Sex-Standardized UPSIT Percentile by Self-Reported Loss of Smell or Taste **eFigure 2.** Proportions of Participants Answering Each UPSIT Question Correctly by Infection Status and self-Report, Among Participants With Abnormal UPSIT Scores **eFigure 3.** Proportions of Participants Answering Each UPSIT Odor-Specific Question Correctly by Infection Status, Self-Reported Loss, and UPSIT Score Among All Participants **eFigure 4.** Clusters of Smell Loss Patterns Among Infected Participants With Self-Reported Loss and Abnormal UPSIT Scores **eFigure 5.** Distribution of Overall UPSIT Score by Cluster Among Infected Participants With Self-Reported Loss and Abnormal UPSIT Scores **eTable 1.** Demographics of Participants Enrolled as Infected by Change or Loss in Smell or Taste, Stratified by Receipt of UPSIT **eTable 2.** Age- and Sex-Standardized UPSIT Percentile by Self-Reported Loss of Smell or Taste, Among Infected Participants Overall, and Stratified by Age, Sex, and Race and Ethnicity **eTable 3.** UPSIT Findings by Cluster Among Infected Participants With Self-Reported Loss and Abnormal UPSIT Scores

Supplement 2.
Nonauthor Collaborators

Supplement 3.
Data Sharing Statement
