# Supplementary material for: Olfactory Dysfunction After SARS-CoV-2 Infection in the RECOVER Adult Cohort
Source: JAMA Netw Open. 2025 Sep 25;8(9):e2533815. doi: 10.1001/jamanetworkopen.2025.33815 (PMC12464792; doi:10.1001/jamanetworkopen.2025.33815)
Supplement: Supplement 1. — eFigure 1. Distribution of Age- and Sex-Standardized UPSIT Percentile by Self-Reported Loss of Smell or Taste eFigure 2. Proportions of Participants Answering Each UPSIT Question Correctly by Infection Status and self-Report, Among Participants With Abnormal UPSIT Scores eFigure 3. Proportions of Participants Answering Each UPSIT Odor-Specific Question Correctly by Infection Status, Self-Reported Loss, and UPSIT Score Among All Participants eFigure 4. Clusters of Smell Loss Patterns Among Infected Participants With Self-Reported Loss and Abnormal UPSIT Scores eFigure 5. Distribution of Overall UPSIT Score by Cluster Among Infected Participants With Self-Reported Loss and Abnormal UPSIT Scores eTable 1. Demographics of Participants Enrolled as Infected by Change or Loss in Smell or Taste, Stratified by Receipt of UPSIT eTable 2. Age- and Sex-Standardized UPSIT Percentile by Self-Reported Loss of Smell or Taste, Among Infected Participants Overall, and Stratified by Age, Sex, and Race and Ethnicity eTable 3. UPSIT Findings by Cluster Among Infected Participants With Self-Reported Loss and Abnormal UPSIT Scores [file jamanetwopen-e2533815-s001.pdf]

Supplementary Online Content

Horwitz LI, Becker JH, Huang W, et al; Researching COVID to Enhance Recovery Consortium. Olfactory dysfunction after SARS-CoV-2 in the RECOVER adult cohort. *JAMA Netw Open.* 2025;8(9):e2533815. doi:10.1001/jamanetworkopen.2025.33815

eFigure 1. Distribution of Age- and Sex-Standardized UPSIT Percentile by Self-Reported Loss of Smell or Taste
eFigure 2. Proportions of Participants Answering Each UPSIT Question Correctly by Infection Status and self-Report, Among Participants With Abnormal UPSIT Scores
eFigure 3. Proportions of Participants Answering Each UPSIT Odor-Specific Question Correctly by Infection Status, Self-Reported Loss, and UPSIT Score Among All Participants
eFigure 4. Clusters of Smell Loss Patterns Among Infected Participants With Self-Reported Loss and Abnormal UPSIT Scores
eFigure 5. Distribution of Overall UPSIT Score by Cluster Among Infected Participants With Self-Reported Loss and Abnormal UPSIT Scores
eTable 1. Demographics of Participants Enrolled as Infected by Change or Loss in Smell or Taste, Stratified by Receipt of UPSIT
eTable 2. Age- and Sex-Standardized UPSIT Percentile by Self-Reported Loss of Smell or Taste, Among Infected Participants Overall, and Stratified by Age, Sex, and Race and Ethnicity
eTable 3. UPSIT Findings by Cluster Among Infected Participants With Self-Reported Loss and Abnormal UPSIT Scores

This supplementary material has been provided by the authors to give readers additional information about their work.

© 2025 Horwitz LI et al. *JAMA Network Open*.

SUPPLEMENT TABLES AND FIGURES

eFigure 1. Distribution of Age- and Sex-Standardized UPSIT Percentile by Self-Reported Loss of Smell or Taste

A. All infected participants

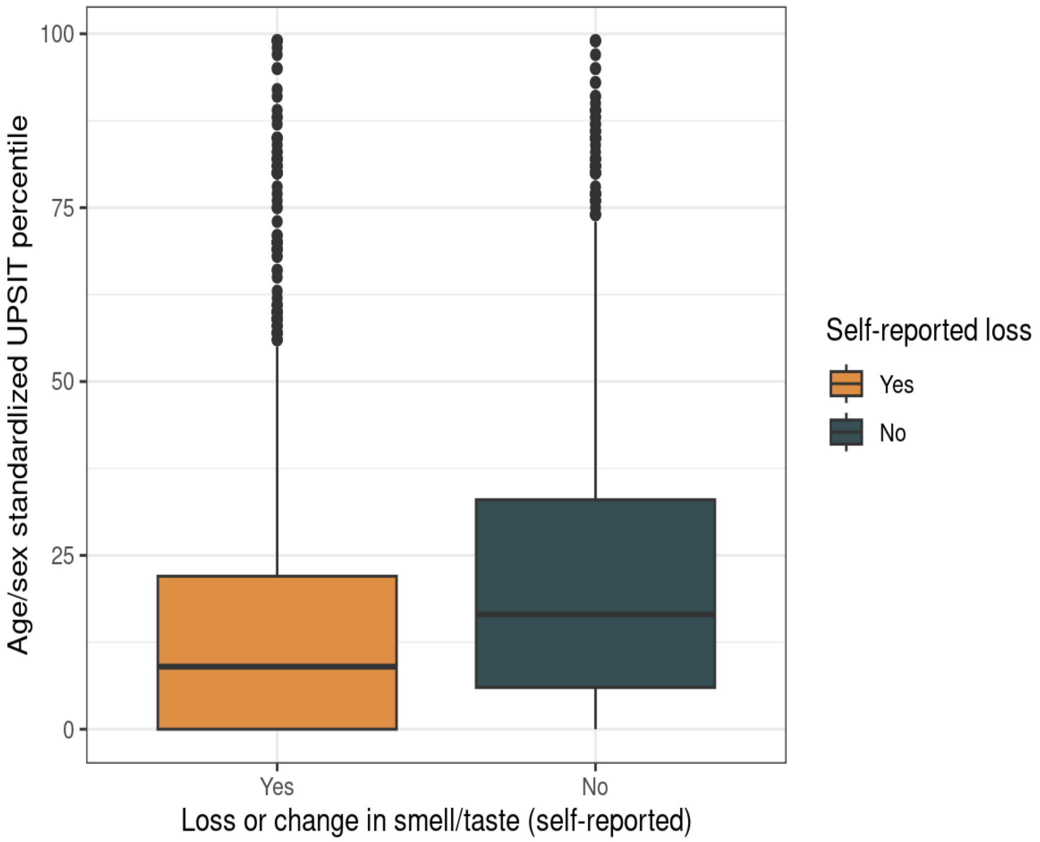

© 2025 Horwitz LI et al. *JAMA Network Open*.

B. Infected participants, by age

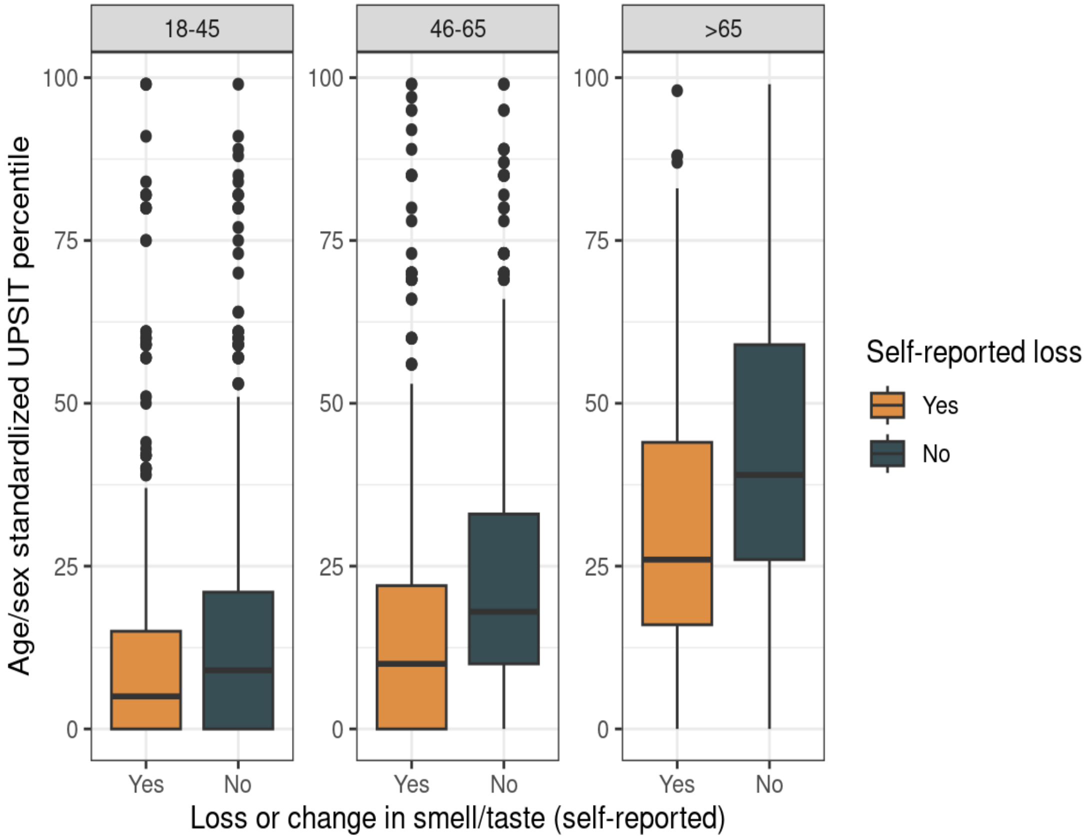

© 2025 Horwitz LI et al. *JAMA Network Open*.

C. Infected participants, by sex

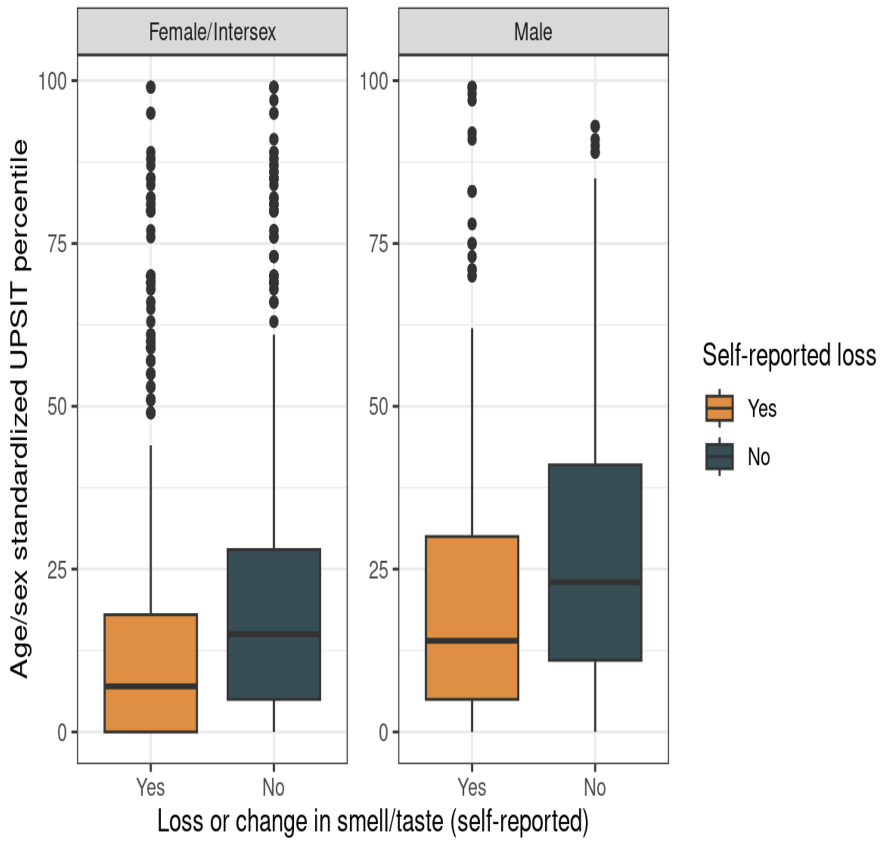

© 2025 Horwitz LI et al. *JAMA Network Open*.

D. Infected participants, by race/ethnicity

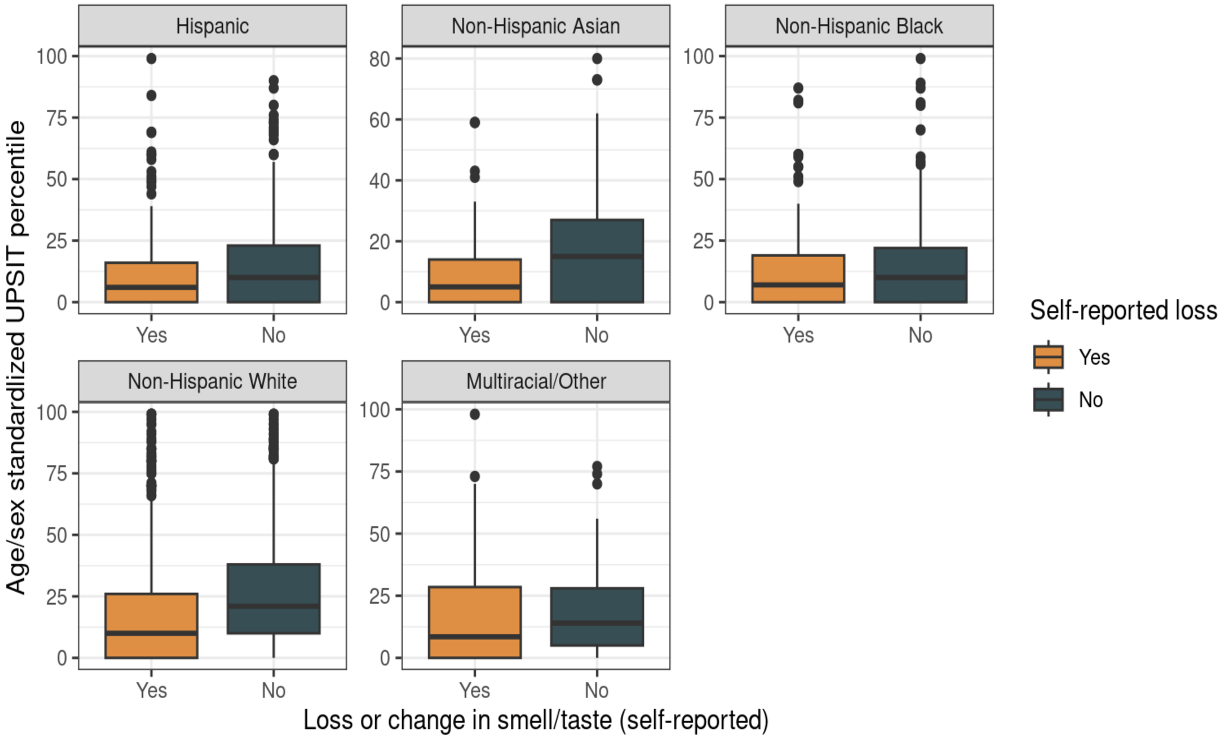

© 2025 Horwitz LI et al. *JAMA Network Open*.

eFigure 2. Proportions of Participants Answering Each UPSIT Question Correctly by Infection Status and self-Report, Among Participants With Abnormal UPSIT Scores

Pleasant Smells	Infection Status	Symptom Reported	Proportion of Correct Answers	n/total N
Grape	Infected	Yes	0.81 (0.79 to 0.83)	899/1111
	Infected	No	0.86 (0.84 to 0.88)	885/1031
	Uninfected	No	0.90 (0.86 to 0.93)	301/336
Cherry	Infected	Yes	0.83 (0.80 to 0.85)	919/1111
	Infected	No	0.90 (0.88 to 0.92)	929/1031
	Uninfected	No	0.88 (0.84 to 0.91)	294/336
Root beer	Infected	Yes	0.80 (0.77 to 0.82)	887/1111
	Infected	No	0.90 (0.88 to 0.91)	924/1031
	Uninfected	No	0.87 (0.84 to 0.91)	293/336
Wintergreen	Infected	Yes	0.80 (0.77 to 0.82)	886/1111
	Infected	No	0.87 (0.85 to 0.89)	896/1031
	Uninfected	No	0.84 (0.80 to 0.88)	282/336
Fruit punch	Infected	Yes	0.70 (0.68 to 0.73)	781/1111
	Infected	No	0.84 (0.82 to 0.86)	868/1031
	Uninfected	No	0.81 (0.76 to 0.85)	271/336
Strawberry	Infected	Yes	0.71 (0.69 to 0.74)	792/1111
	Infected	No	0.80 (0.77 to 0.82)	823/1031
	Uninfected	No	0.78 (0.74 to 0.82)	262/336
Pineapple	Infected	Yes	0.68 (0.66 to 0.71)	760/1111
	Infected	No	0.76 (0.74 to 0.79)	785/1031
	Uninfected	No	0.77 (0.73 to 0.82)	259/336
Chocolate	Infected	Yes	0.75 (0.72 to 0.77)	828/1111
	Infected	No	0.79 (0.76 to 0.81)	813/1031
	Uninfected	No	0.76 (0.71 to 0.80)	254/336
Cinnamon	Infected	Yes	0.63 (0.60 to 0.66)	700/1111
	Infected	No	0.72 (0.69 to 0.75)	740/1031
	Uninfected	No	0.74 (0.70 to 0.79)	250/336
Bubble gum	Infected	Yes	0.60 (0.57 to 0.63)	668/1111
	Infected	No	0.70 (0.67 to 0.73)	723/1031
	Uninfected	No	0.69 (0.64 to 0.74)	231/336
Watermelon	Infected	Yes	0.54 (0.52 to 0.57)	605/1111
	Infected	No	0.67 (0.64 to 0.70)	687/1031
	Uninfected	No	0.67 (0.62 to 0.72)	226/336
Banana	Infected	Yes	0.50 (0.47 to 0.53)	558/1111
	Infected	No	0.58 (0.55 to 0.61)	597/1031
	Uninfected	No	0.56 (0.51 to 0.61)	188/336
Orange	Infected	Yes	0.45 (0.42 to 0.48)	502/1111
	Infected	No	0.50 (0.47 to 0.53)	518/1031
	Uninfected	No	0.50 (0.45 to 0.56)	169/336
Lime	Infected	Yes	0.40 (0.37 to 0.43)	447/1111
	Infected	No	0.38 (0.35 to 0.40)	387/1031
	Uninfected	No	0.39 (0.33 to 0.44)	130/336
Lemon	Infected	Yes	0.32 (0.29 to 0.35)	358/1111
	Infected	No	0.33 (0.30 to 0.36)	340/1031
	Uninfected	No	0.32 (0.27 to 0.37)	108/336

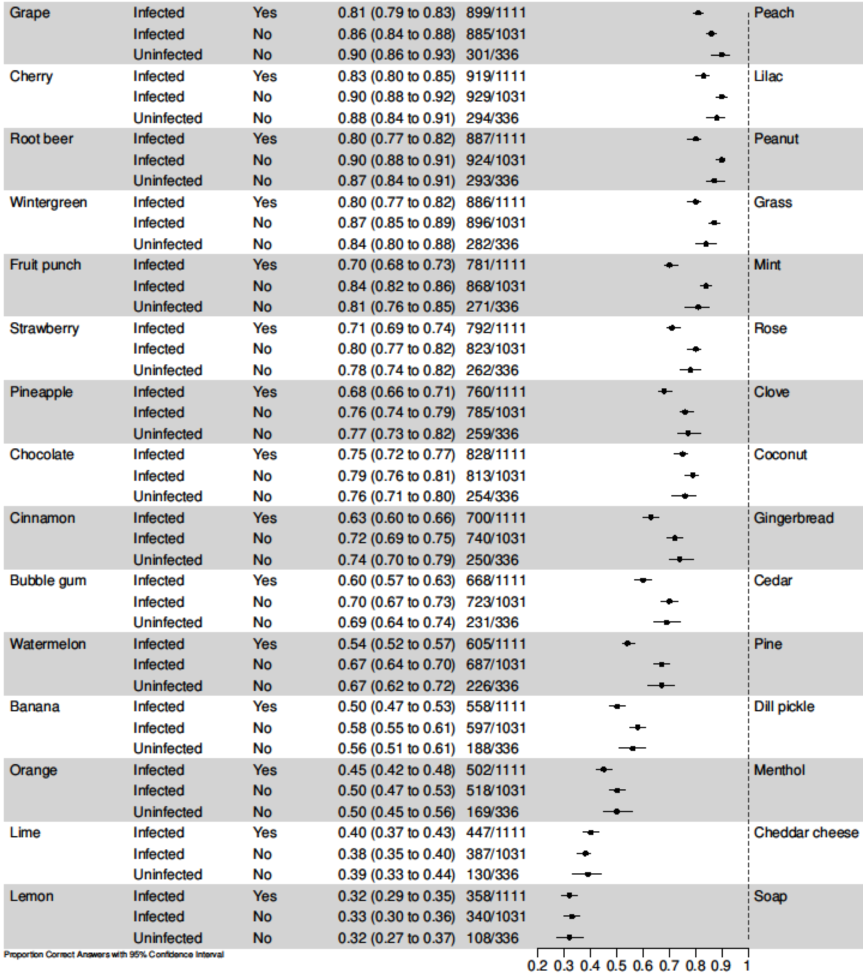

Neutral Smells	Proportion of Correct Answers	n/total N
Peach	0.86 (0.84 to 0.88)	956/1111
	0.91 (0.90 to 0.93)	941/1031
	0.92 (0.89 to 0.95)	308/336
Lilac	0.83 (0.80 to 0.85)	919/1111
	0.90 (0.88 to 0.91)	924/1031
	0.89 (0.86 to 0.93)	300/336
Peanut	0.83 (0.81 to 0.85)	922/1111
	0.89 (0.87 to 0.91)	918/1031
	0.88 (0.85 to 0.92)	297/336
Grass	0.74 (0.71 to 0.77)	822/1111
	0.87 (0.84 to 0.89)	892/1031
	0.87 (0.84 to 0.91)	293/336
Mint	0.79 (0.77 to 0.82)	879/1111
	0.88 (0.86 to 0.90)	907/1031
	0.85 (0.82 to 0.89)	287/336
Rose	0.79 (0.76 to 0.81)	874/1111
	0.89 (0.87 to 0.91)	919/1031
	0.85 (0.82 to 0.89)	287/336
Clove	0.69 (0.66 to 0.71)	764/1111
	0.81 (0.78 to 0.83)	832/1031
	0.83 (0.79 to 0.87)	280/336
Coconut	0.68 (0.66 to 0.71)	760/1111
	0.77 (0.75 to 0.80)	798/1031
	0.76 (0.71 to 0.80)	254/336
Gingerbread	0.68 (0.66 to 0.71)	760/1111
	0.75 (0.73 to 0.78)	778/1031
	0.73 (0.68 to 0.78)	245/336
Cedar	0.59 (0.56 to 0.62)	655/1111
	0.67 (0.65 to 0.70)	695/1031
	0.71 (0.66 to 0.75)	237/336
Pine	0.63 (0.60 to 0.65)	696/1111
	0.74 (0.72 to 0.77)	765/1031
	0.71 (0.67 to 0.76)	240/336
Dill pickle	0.66 (0.64 to 0.69)	738/1111
	0.72 (0.69 to 0.75)	744/1031
	0.70 (0.65 to 0.75)	235/336
Menthol	0.51 (0.48 to 0.54)	562/1111
	0.57 (0.54 to 0.60)	583/1031
	0.60 (0.55 to 0.65)	201/336
Cheddar cheese	0.64 (0.61 to 0.67)	709/1111
	0.68 (0.66 to 0.71)	705/1031
	0.59 (0.54 to 0.64)	199/336
Soap	0.54 (0.52 to 0.57)	605/1111
	0.60 (0.57 to 0.63)	620/1031
	0.59 (0.54 to 0.64)	198/336

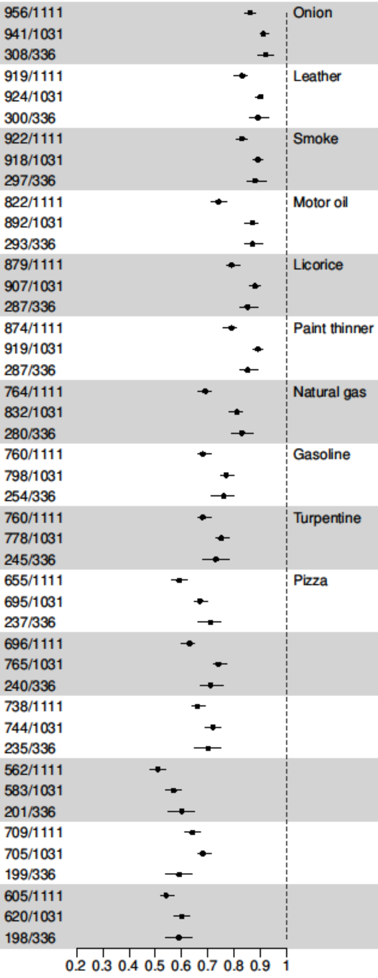

Unpleasant Smells	Proportion of Correct Answers	n/total N
Onion	0.93 (0.91 to 0.94)	1029/1111
	0.97 (0.95 to 0.98)	996/1031
	0.96 (0.94 to 0.98)	323/336
Leather	0.82 (0.80 to 0.84)	911/1111
	0.93 (0.91 to 0.94)	956/1031
	0.90 (0.86 to 0.93)	301/336
Smoke	0.77 (0.74 to 0.79)	850/1111
	0.86 (0.83 to 0.88)	882/1031
	0.85 (0.81 to 0.89)	286/336
Motor oil	0.77 (0.74 to 0.79)	850/1111
	0.84 (0.82 to 0.87)	869/1031
	0.82 (0.78 to 0.86)	275/336
Licorice	0.69 (0.67 to 0.72)	769/1111
	0.83 (0.80 to 0.85)	852/1031
	0.82 (0.78 to 0.86)	275/336
Paint thinner	0.73 (0.71 to 0.76)	813/1111
	0.79 (0.76 to 0.81)	811/1031
	0.79 (0.75 to 0.84)	266/336
Natural gas	0.70 (0.68 to 0.73)	781/1111
	0.80 (0.78 to 0.83)	829/1031
	0.78 (0.73 to 0.82)	261/336
Gasoline	0.63 (0.61 to 0.66)	704/1111
	0.66 (0.63 to 0.69)	680/1031
	0.64 (0.59 to 0.69)	215/336
Turpentine	0.45 (0.42 to 0.48)	504/1111
	0.51 (0.48 to 0.54)	522/1031
	0.48 (0.43 to 0.53)	161/336
Pizza	0.43 (0.40 to 0.46)	475/1111
	0.46 (0.43 to 0.49)	470/1031
	0.42 (0.36 to 0.47)	140/336

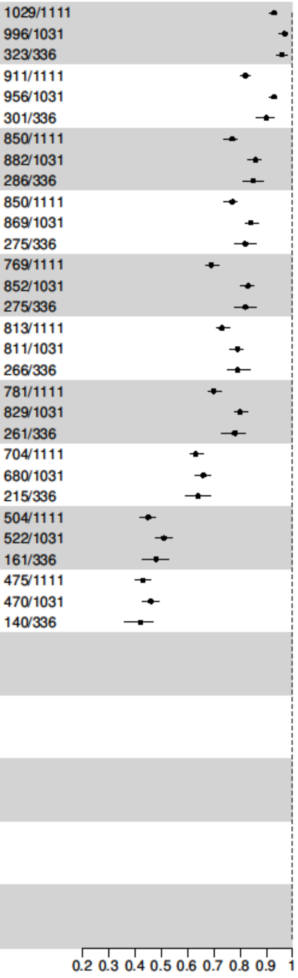

Proportion Correct Answers with 95% Confidence Interval

© 2025 Horwitz LI et al. *JAMA Network Open*.

eFigure 3. Proportions of Participants Answering Each UPSIT Odor-Specific Question Correctly by Infection Status, Self-Reported Loss, and UPSIT Score Among All Participants

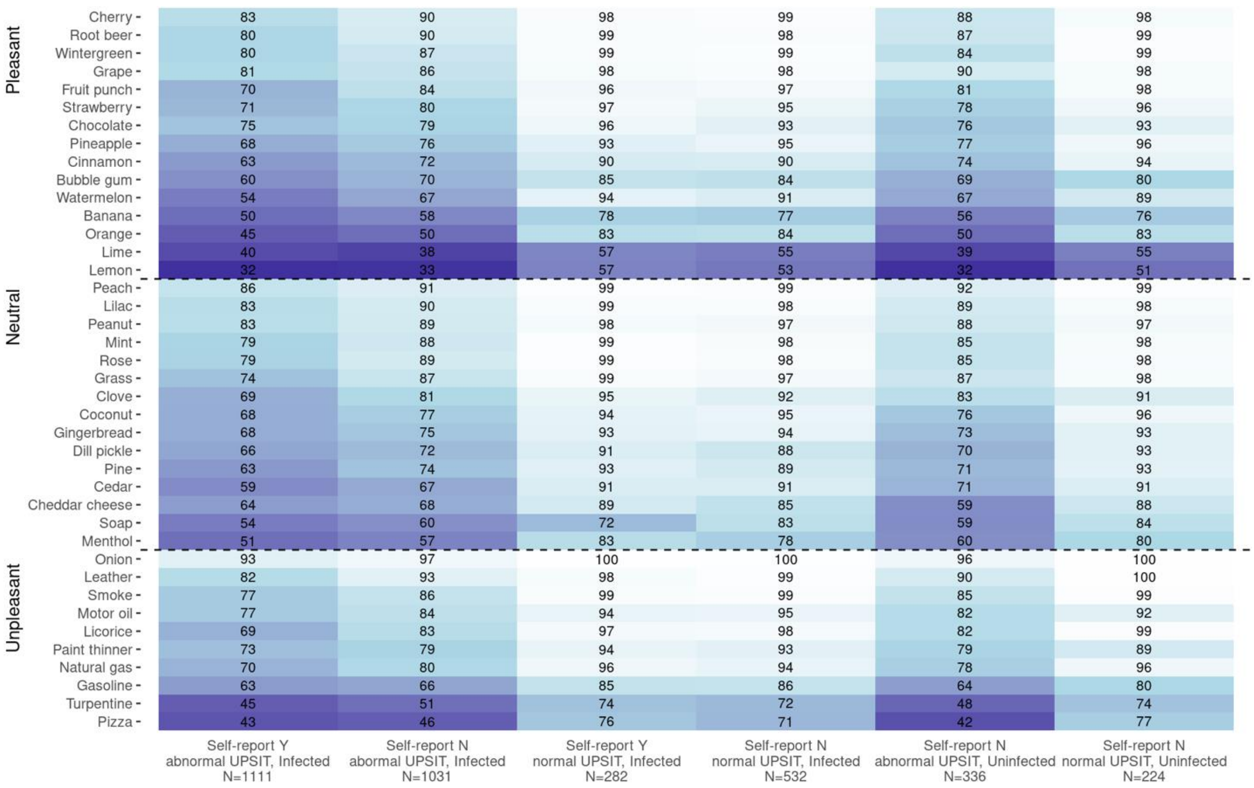

Category	Odor	Self-report Y abnormal UPSIT, Infected N=1111	Self-report N abnormal UPSIT, Infected N=1031	Self-report Y normal UPSIT, Infected N=282	Self-report N normal UPSIT, Infected N=532	Self-report N abnormal UPSIT, Uninfected N=336	Self-report N normal UPSIT, Uninfected N=224
Pleasant	Cherry	83	90	98	99	88	98
	Root beer	80	90	99	98	87	99
	Wintergreen	80	87	99	99	84	99
	Grape	81	86	98	98	90	98
	Fruit punch	70	84	96	97	81	98
	Strawberry	71	80	97	95	78	96
	Chocolate	75	79	96	93	76	93
	Pineapple	68	76	93	95	77	96
	Cinnamon	63	72	90	90	74	94
	Bubble gum	60	70	85	84	69	80
	Watermelon	54	67	94	91	67	89
	Banana	50	58	78	77	56	76
	Orange	45	50	83	84	50	83
	Lime	40	38	57	55	39	55
	Lemon	32	33	57	53	32	51
Neutral	Peach	86	91	99	99	92	99
	Lilac	83	90	99	98	89	98
	Peanut	83	89	98	97	88	97
	Mint	79	88	99	98	85	98
	Rose	79	89	99	98	85	98
	Grass	74	87	99	97	87	98
	Clove	69	81	95	92	83	91
	Coconut	68	77	94	95	76	96
	Gingerbread	68	75	93	94	73	93
	Dill pickle	66	72	91	88	70	93
	Pine	63	74	93	89	71	93
	Cedar	59	67	91	91	71	91
	Cheddar cheese	64	68	89	85	59	88
	Soap	54	60	72	83	59	84
	Menthol	51	57	83	78	60	80
Unpleasant	Onion	93	97	100	100	96	100
	Leather	82	93	98	99	90	100
	Smoke	77	86	99	99	85	99
	Motor oil	77	84	94	95	82	92
	Licorice	69	83	97	98	82	99
	Paint thinner	73	79	94	93	79	89
	Natural gas	70	80	96	94	78	96
	Gasoline	63	66	85	86	64	80
	Turpentine	45	51	74	72	48	74
	Pizza	43	46	76	71	42	77

© 2025 Horwitz LI et al. *JAMA Network Open*.

eFigure 4. Clusters of Smell Loss Patterns Among Infected Participants With Self-Reported Loss and Abnormal UPSIT Scores

A. Percent correct odor identification, by cluster

	Odor	Cluster 1 N=358	Cluster 2 N=389	Cluster 3 N=225	Cluster 4 N=139
Pleasant	Cherry	93	94	73	39
	Grape	89	94	70	42
	Root beer	92	90	73	31
	Wintergreen	91	89	72	35
	Chocolate	81	84	69	42
	Strawberry	81	83	59	33
	Fruit punch	88	85	51	16
	Pineapple	78	83	53	29
	Cinnamon	75	75	44	29
	Bubble gum	70	70	56	15
	Watermelon	57	72	41	21
	Banana	54	57	46	29
	Orange	47	59	31	26
	Lime	38	49	34	31
	Lemon	31	38	28	27
Neutral	Peach	96	95	85	39
	Lilac	93	95	71	40
	Peanut	89	90	84	44
	Mint	89	90	63	47
	Rose	91	90	68	30
	Grass	84	86	61	35
	Clove	76	78	65	32
	Coconut	79	81	55	28
	Gingerbread	73	80	61	37
	Dill pickle	80	78	52	20
	Cheddar cheese	74	69	57	33
	Pine	73	74	42	39
	Cedar	72	74	36	23
	Soap	63	59	45	34
	Menthol	55	54	49	33
Unpleasant	Onion	98	99	92	63
	Leather	89	95	71	45
	Motor oil	87	85	72	35
	Smoke	90	91	57	33
	Paint thinner	89	71	74	38
	Natural gas	79	82	58	35
	Licorice	87	80	44	35
	Gasoline	67	67	66	40
	Turpentine	100	0	47	29
	Pizza	45	54	32	22

B. Kmeans consensus cluster fit

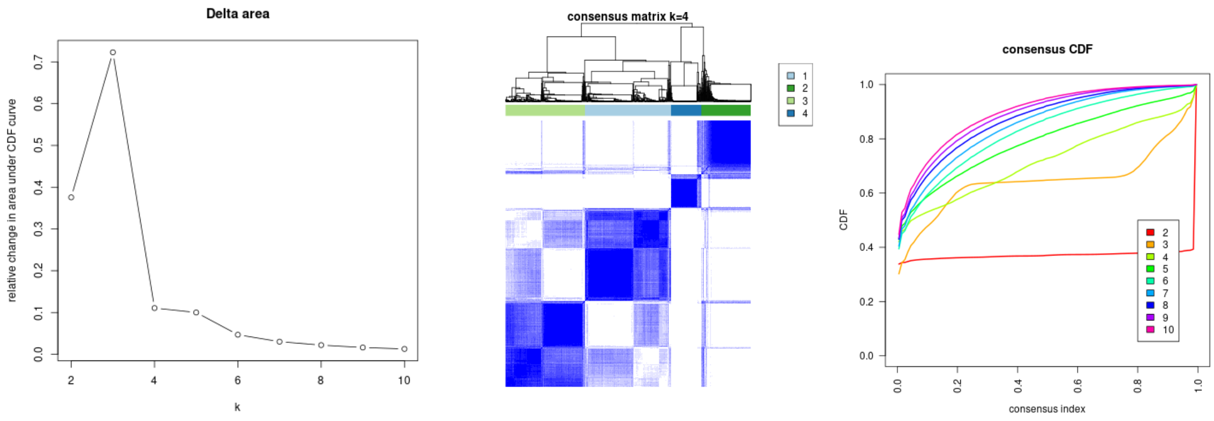

© 2025 Horwitz LI et al. *JAMA Network Open*.

eFigure 5. Distribution of Overall UPSIT Score by Cluster Among Infected Participants With Self-Reported Loss and Abnormal UPSIT Scores

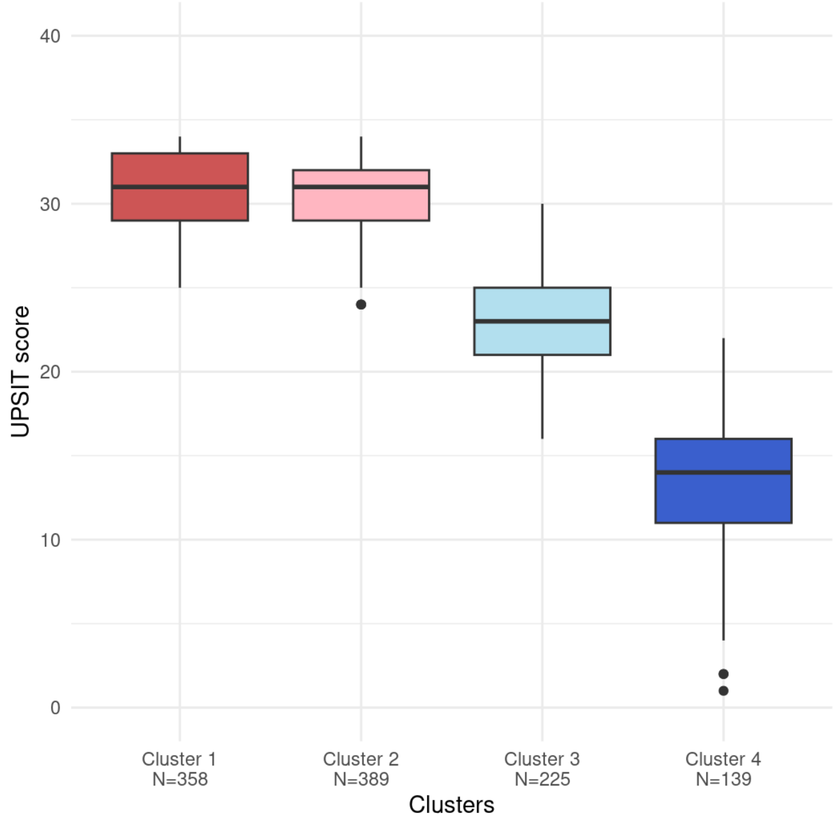

© 2025 Horwitz LI et al. *JAMA Network Open*.

SUPPLEMENT TABLES

eTable 1. Demographics of Participants Enrolled as Infected by Change or Loss in Smell or Taste, Stratified by Receipt of UPSIT
A Reporting Change or Loss in Smell or Taste

**Characteristic**	**Completed UPSIT N = 1,361**	**Did not Complete UPSIT N = 667**
Age at index		
Mean (SD)	47.4 (14.7)	46.3 (15.2)
Median (IQR)	47.1 (35.1, 58.7)	45.3 (33.4, 57.5)
Age Category at index		
18-45	612 (45%)	327 (49%)
46-65	568 (42%)	257 (39%)
>65	181 (13%)	83 (12%)
Sex assigned at birth		
Female/Intersex	1,053 (78%)	516 (78%)
Male	304 (22%)	148 (22%)
Missing	4	3
Race/Ethnicity		
Hispanic	286 (21%)	129 (20%)
Non-Hispanic Asian	41 (3.0%)	22 (3.3%)
Non-Hispanic Black	162 (12%)	118 (18%)
Non-Hispanic White	812 (60%)	346 (53%)
Mixed race/Other	51 (3.8%)	42 (6.4%)
Missing	9	10
Enrollment cohort and era		
Pre-Omicron	787 (58%)	306 (46%)
Acute, Omicron	268 (20%)	163 (24%)
Post-Acute, Omicron	306 (22%)	198 (30%)
Vaccinated at first infection		
Date of last dose unknown	26 (1.9%)	22 (3.4%)
Fully vaccinated	596 (44%)	323 (50%)
Partially vaccinated	26 (1.9%)	19 (2.9%)
Unvaccinated	695 (52%)	286 (44%)
Missing	18	17
Acute Hospitalization		
Not Hospitalized	1,145 (88%)	544 (88%)
Hospitalized during acute phase	162 (12%)	72 (12%)
Missing	54	51
Household Income		

© 2025 Horwitz LI et al. *JAMA Network Open*.

Characteristic	Completed UPSIT N = 1,361	Did not Complete UPSIT N = 667
<$25,000	214 (17%)	130 (22%)
$25,000-$49,999	234 (19%)	108 (18%)
>$50,000	780 (64%)	349 (59%)
Missing	133	80
Rural/Urban		
Not rural participant	1,258 (92%)	624 (94%)
Rural participant	103 (7.6%)	43 (6.4%)
Education		
Bachelors / Advanced degree	746 (57%)	348 (55%)
High school / GED / Some college / vocational / technical	558 (43%)	285 (45%)
Missing	57	34

© 2025 Horwitz LI et al. *JAMA Network Open*.

B Never Reporting Any Change or Loss in Smell or Taste

**Characteristic**	**Completed UPSIT N = 520**	**Did not complete UPSIT N = 1193**
Age at index		
Mean (SD)	50.6 (15.2)	48.4 (15.9)
Median (IQR)	52.4 (37.9, 62.5)	48.4 (34.7, 61.3)
Age Category at index		
18-45	197 (38%)	541 (45%)
46-65	229 (44%)	432 (36%)
>65	94 (18%)	220 (18%)
Sex assigned at birth		
Female/Intersex	344 (66%)	853 (72%)
Male	176 (34%)	334 (28%)
Missing	0	6
Race/Ethnicity		
Hispanic	69 (13%)	135 (11%)
Non-Hispanic Asian	32 (6.2%)	85 (7.2%)
Non-Hispanic Black	82 (16%)	163 (14%)
Non-Hispanic White	314 (61%)	754 (64%)
Mixed race/Other	21 (4.1%)	44 (3.7%)
Missing	2	12
Enrollment cohort and era		
Pre-Omicron	106 (20%)	210 (18%)
Acute, Omicron	234 (45%)	556 (47%)
Post-Acute, Omicron	180 (35%)	427 (36%)
Vaccinated at first infection		
Date of last dose unknown	9 (1.8%)	35 (3.0%)
Fully vaccinated	423 (83%)	984 (85%)
Partially vaccinated	9 (1.8%)	17 (1.5%)
Unvaccinated	67 (13%)	122 (11%)
Missing	12	35
Household Income		
<$25,000	93 (20%)	173 (16%)
$25,000-$49,999	60 (13%)	132 (12%)
>$50,000	316 (67%)	780 (72%)
Missing	51	108
Rural/Urban		
Not rural participant	501 (96%)	1,149 (96%)
Rural participant	19 (3.7%)	44 (3.7%)
Education		
Bachelors / Advanced degree	356 (73%)	853 (74%)

© 2025 Horwitz LI et al. *JAMA Network Open*.

Characteristic	Completed UPSIT N = 520	Did not complete UPSIT N = 1193
High school / GED / Some college / vocational / technical	134 (27%)	297 (26%)
High school / GED / Some college / vocational / technical	134 (27%)	297 (26%)
Missing	30	43

© 2025 Horwitz LI et al. *JAMA Network Open*.

eTable 2. Age- and Sex-Standardized UPSIT Percentile by Self-Reported Loss of Smell or Taste, Among Infected Participants Overall, and Stratified by Age, Sex, and Race and Ethnicity

	**Self-reported loss of or change in smell or taste**	
**UPSIT age/sex percentiles, median (IQR)**	**Yes**	**No**
Overall Infected	9 (0, 22)	17 (6, 33)
Sex		
Female/Intersex	7 (0, 18)	15 (5, 28)
Male	14 (5, 30)	23 (11, 41)
Age		
Age 18-45	5 (0, 15)	9 (0, 21)
Age 46-65,	10 (0, 22)	18 (10, 33)
Age >65	26 (16, 44)	39 (26, 59)
Race/ethnicity		
Hispanic	6 (0, 16)	10 (0, 23)
Non-Hispanic Asian	5 (0, 14)	15 (0, 27)
Non-Hispanic Black	7 (0, 19)	10 (0, 22)
Non-Hispanic White	10 (0, 26)	21 (10, 38)
Multiracial/Other	9 (0, 29)	14 (5, 28)

eTable 3. UPSIT Findings by Cluster Among Infected Participants With Self-Reported Loss and Abnormal UPSIT Scores

**Characteristic**	**Cluster 1 N = 358**	**Cluster 2 N = 389**	**Cluster 3 N = 225**	**Cluster 4 N = 139**	**Overall N = 1,111**
Microsmia status, n (%)					
Mild microsmia	212 (59%)	207 (53%)	1 (0.4%)	0 (0%)	420 (38%)
Moderate microsmia	145 (41%)	173 (44%)	41 (18%)	0 (0%)	359 (32%)
Severe microsmia	1 (0.3%)	9 (2.3%)	177 (79%)	7 (5.0%)	194 (17%)
Anosmia	0 (0%)	0 (0%)	6 (2.7%)	121 (87%)	127 (11%)
Score <6	0 (0%)	0 (0%)	0 (0%)	11 (7.9%)	11 (1.0%)
UPSIT score					
Mean (SD)	30.8 (2.2)	30.4 (2.4)	23.1 (2.6)	13.3 (4.3)	26.9 (6.5)
Median (IQR)	31.0 (29.0, 33.0)	31.0 (29.0, 32.0)	23.0 (21.0, 25.0)	14.0 (11.0, 16.0)	29.0 (24.0, 32.0)

© 2025 Horwitz LI et al. *JAMA Network Open*.
