# Supplementary material for: Olfactory Dysfunction After SARS-CoV-2 Infection in the RECOVER Adult Cohort
Source: JAMA Netw Open. 2025 Sep 25;8(9):e2533815. doi: 10.1001/jamanetworkopen.2025.33815 (PMC12464792; doi:10.1001/jamanetworkopen.2025.33815)
Supplement: Supplement 3. — Data Sharing Statement [file jamanetwopen-e2533815-s003.pdf]

Data Sharing Statement

Horwitz. Olfactory Dysfunction After SARS-CoV-2 in the RECOVER Adult Cohort. *JAMA Netw Open*. Published September 25, 2025. doi:10.1001/jamanetworkopen.2025.33815

Data
Data available: Yes
Data types: Deidentified participant data
How to access data: The data dictionary from the RECOVER Adult Cohort Observational Study is published on the RECOVER website (https://recovercovid.org/data). NHLBI has undertaken a significant effort to release harmonized data from all RECOVER observational cohort studies to the public via their BioData Catalyst platform (https://biodatacatalyst.nhlbi.nih.gov/). Datasets include participant-level data collected for the present study, as well as the technical infrastructure and analytic tools to evaluate the results and conclusions from this study.
When available: With publication

Supporting Documents
Document types: None

Additional Information
Who can access the data: Researchers whose proposed use of the data has been approved
Types of analyses: For a specified scientific purpose
Mechanisms of data availability: After approval of a proposal and with a signed data access agreement
